# Development and validation of the information needs questionnaire for differentiated thyroid cancer patient with radioactive iodine therapy (INQ‐DTC)

**DOI:** 10.1002/nop2.925

**Published:** 2021-05-18

**Authors:** Jing Zhang, Limei Li, Dongling Liu, Tingting Zhu, Qiaoqiao Gao, Hui Chen, Ling Ma, Jiayin Li, Zichen Wang

**Affiliations:** ^1^ School of Nursing and Health Zhengzhou University Zhengzhou China; ^2^ The First Affiliated Hospital of Zhengzhou University Zhengzhou China; ^3^ The First Affiliated Hospital of Henan University of Chinese Medicine Zhengzhou China

**Keywords:** information needs, scale, thyroid neoplasms, validity

## Abstract

**Aims:**

This study aimed to develop and validate the psychometric properties of Information Needs Questionnaire for Differentiated Thyroid Cancer (INQ‐DTC) in DTC patients with radioactive iodine (RAI) therapy.

**Design:**

Mixed methods.

**Methods:**

Using qualitative methods, we developed the initial questionnaire from a personal perspective of information needs of 15 patients with DTC. We used a formal Delphi consensus process to help assess the initial questionnaire and provide recommendations for its application. Totally, 230 DTC patients with RAI therapy were selected for the process of validation.

**Results:**

The final version of INQ‐DTC contains 33 items. The total Cronbach's alpha coefficient was 0.945, the total split‐half reliability was 0.822, and the test‐retest value was 0.984 for the overall score. Exploratory factor analysis extracted 5 factors, which could explain 61.86% of the total variance. The Scale‐level content validity index (S‐CVI) was 0.928, and 0.929 for the item‐level content validity index (I‐CVI).

## 1 INTRODUCTION

Thyroid cancer is the fastest‐growing cancer among all solid tumours in recent years (Bray et al., 2018; Chen et al., 2016; Siegel et al., 2019). Differentiated thyroid cancer (DTC) is the most common histological type of thyroid cancer (>90%), and radioactive iodine (RAI) therapy is one of the standard treatments for DTC (Cabanillas et al., 2016; Haugen et al., 2016).

The treatment cycle of DTC patients who had undergone RAI therapy is long and complex (Cabanillas et al., 2016), which including multiple phases (e.g. surgery, hormone withdrawal, RAI therapy and home‐based rehabilitation) (Haugen et al., 2016). To make this lengthy process proceed successfully, patients need to know tremendous information (Morley & Goldfarb, 2015). However, only a few of them indicated that they received the information support (Goldfarb & Casillas, 2014; Morley & Goldfarb, 2015; Sawka et al., 2016). The fact that most of the patients lack of enough information support makes them vulnerable to false information (Kuenzel et al., 2018) and results negative emotions (Heckel et al., 2015; Husson et al., 2013). In addition, information is an important basis for patients to participate in the communication (Heynsbergh et al., 2018) and decision‐making process (Nickel et al., 2018). Evaluating the information need is an important step towards personalized information support (Arraras et al., 2010), patient‐centred care (Ulloa et al., 2015) and health‐related quality of life (Lamers et al., 2016).

Coordinating of multidisciplinary care team to assess the status of the patient information needs provides continuity of health education and pertinent information support for patients are important responsibilities and work content for nurses. And it is also an important requirement of patient‐centred quality care and full life‐cycle care. So understanding and evaluating the information needs of DTC patients has become an important issue for clinical nurses to focus on.

In order for information needs accurate assessment to service these aims, we should consider several aspects as following. Firstly, due to the special nature of RAI therapy, information needs assessment should cover specificity issues for which DTC patients need support, such as low iodine diet and home‐based radiation protection information (Goldfarb & Casillas, 2014; Kim et al., 2010; Morley & Goldfarb, 2015). Secondly, the accuracy and comprehensiveness of information need from the patients should also be taken seriously. Multiple reasons, such as educational level and cultural conservatism, can cause misunderstanding and missing information when patients are describing their situations, so it is important to identify the implicit information need of patients (Owen‐Smith et al., 2010; Zhang et al., 2019).

However, there are few information needs instruments have been developed for DTC patients despite the importance of identifying patient information need. Existing tools could be divided into two types (universal questionnaire and specific questionnaire). The former mostly focuses on the common information needs of a wide variety of cancer patients (Arraras et al., 2010; Dall'Armi et al., 2013; Huang et al., 2003). Given the increasing concern of the specific information need of DTC patients (Sawka et al., 2018), it seems opportune to develop information needs assessment tools that could reflect the specific information needs of DTC patients. Another aspect that should be considered is the reliability and standardization of the tool, existing specific questionnaire is potentially useful but it is not yet validated (Morley & Goldfarb, 2015).

To overcome the limitation of previous information needs assessment tools for DTC patients, we decided to develop the first specific information needs questionnaire for DTC patients in mainland China and systematically assessed the psychometric properties of it. The Information Needs Questionnaire for Differentiated Thyroid Cancer (INQ‐DTC) aims to help to identify areas for information support development which could ultimately improve the individual care and information support received by DTC patients. This article reports the development and validation of INQ‐DTC.

## 2 MATERIALS AND METHODS

**FIGURE 1:**
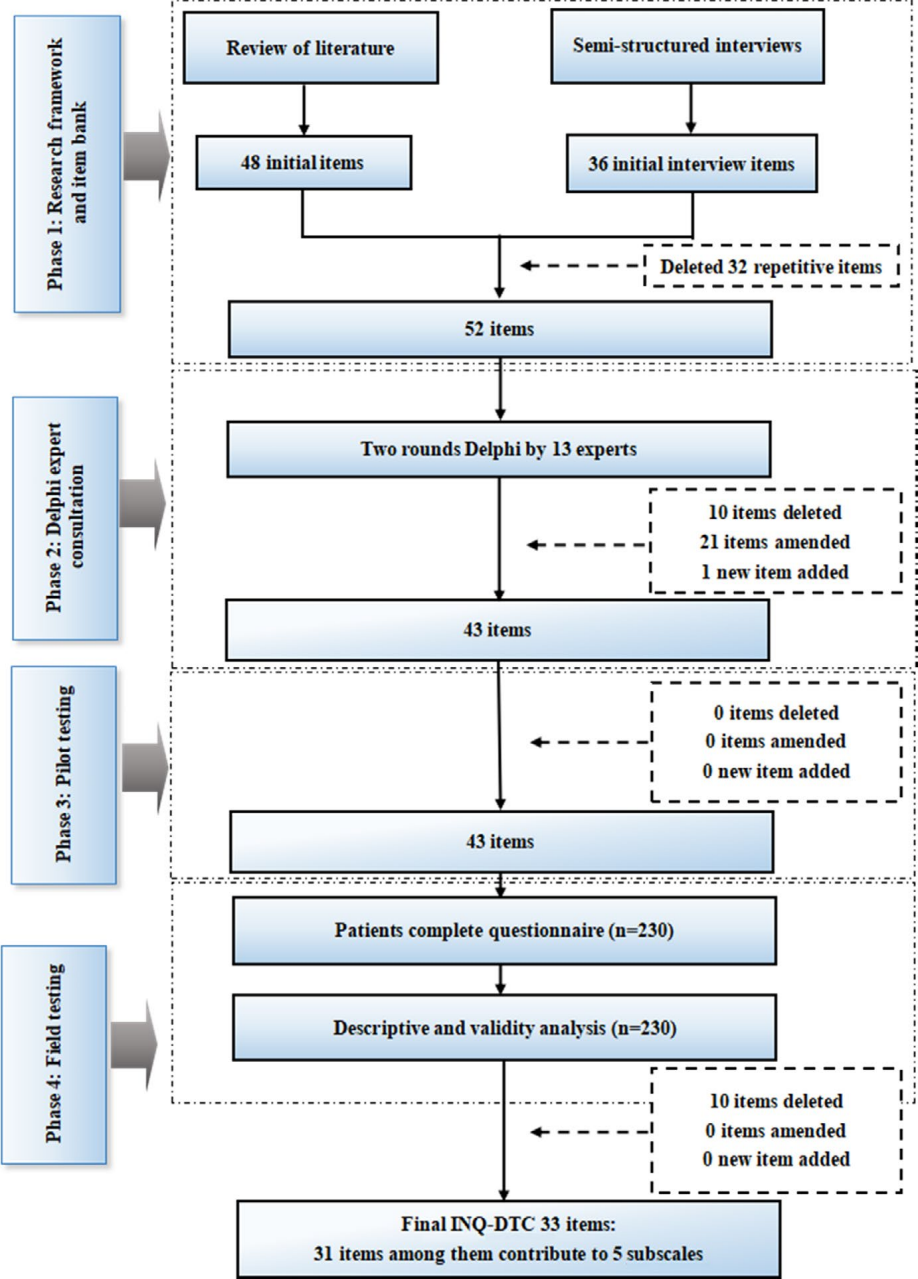
Overview of the INQ‐DTC development process

A mixed methods was undertaken to develop the questionnaire in this study. The STROBE guidelines for the reporting of observational studies were followed (See Supplementary File 1). We performed four phases: phase 1 “research framework and item bank” generated themes of the questionnaire and items based on patient semi‐structured interviews; phase 2 “Delphi expert consultation,” experts confirmed the original version and provided amendments for it; phase 3 “pilot testing” tested the feasibility and acceptability of this tool; followed by phase 4: “field testing” assessed the reliability and validity of the questionnaire using psychometric analysis. We developed the questionnaire in accordance with best practice guidelines in questionnaire development (Figure 1) (Johnson et al., 2011). This study was conducted from November 2017 to March 2019 and approved by the Research Ethical Committee of Zhengzhou University.

### 2.1 Phase 1: research framework and item bank

#### 2.1.1 Research framework and questionnaire themes development

**FIGURE 2:**
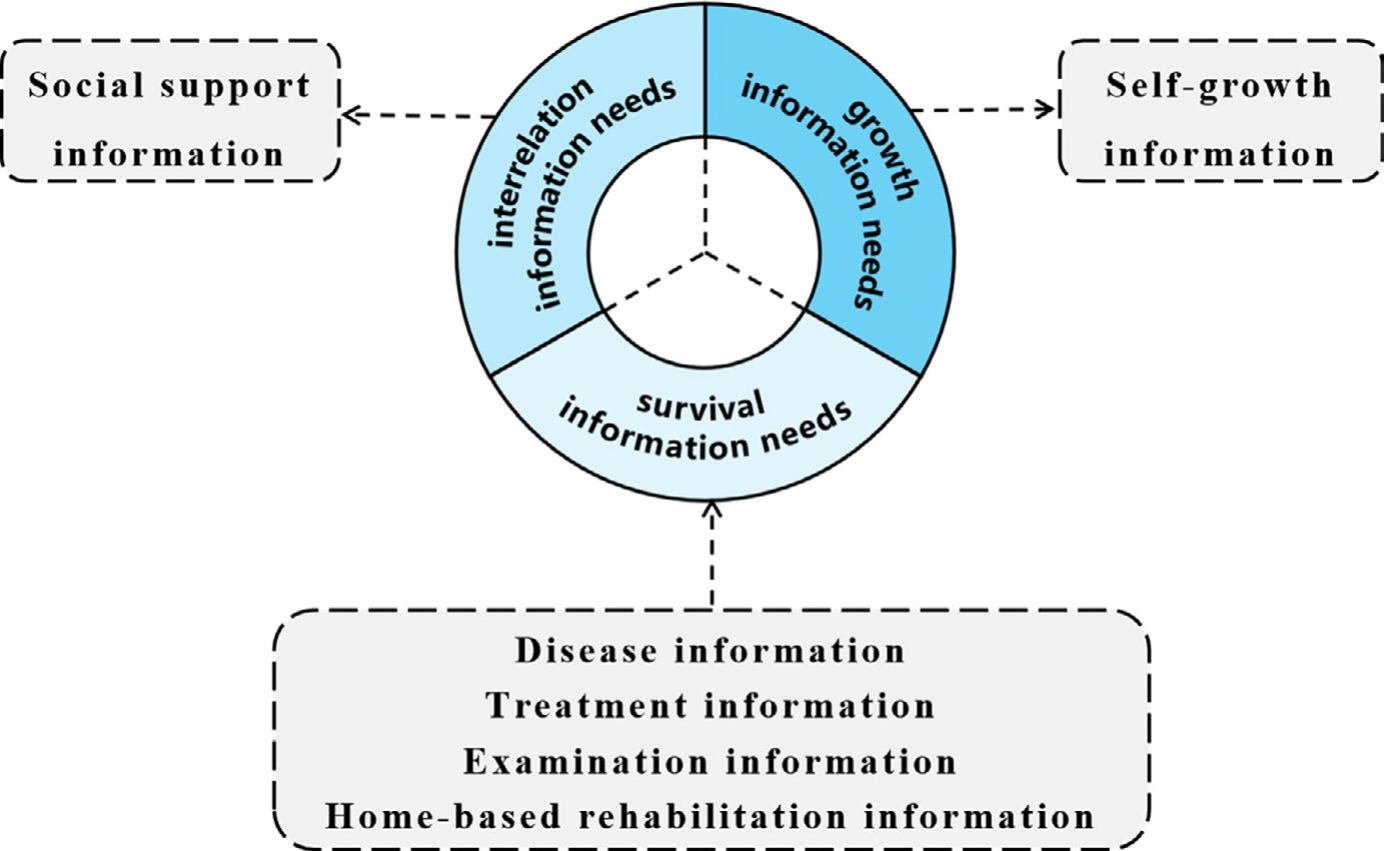
Research framework

Existence Relatedness Growth (ERG) theory (Alderfer, 1969) was used to establish the research framework for the questionnaire, which divided the information needs of DTC patients into three categories: survival information needs, interrelation information needs and growth information needs. In order to develop the themes of the questionnaire, we reviewed existing information needs assessment tools and related literature. Based on the results of literature review, the information needs of DTC patients were divided into six themes: disease information, treatment information, examination information, home‐based rehabilitation information, social support information and self‐growth information. The relationship of ERG theory and questionnaire themes was summarized in Figure 2.

#### 2.1.2 Item/domain generation

We reviewed information needs related tools for cancer. Six valid questionnaires were selected: Supportive Care Needs Survey‐Short Form (Au et al., 2011; Boyes et al., 2009); Cancer Needs Questionnaires‐Short Form (Cossich et al., 2004); Cancer Survivor's Unmet Need (Fang et al., 2018; Hodgkinson et al., 2007); Information Preference Question for Cancer Patients (Huang et al., 2003); European Organization for Research and Treatment of Cancer Quality of Life Group Information Questionnaire (Arraras et al., 2010); Head and Neck Information Needs Questionnaire (Dall'Armi et al., 2013). Based on existing questionnaires and literature, 48 items were extracted.

We interviewed 15 DTC patients with RAI therapy with the aim of supplementing item bank, on the basis of 48 item bank, the initial questionnaire consisting of 52 items was established for the Delphi process. The five‐point Likert scale was used in this questionnaire, “1” = not needed at all; “2” = low degree of need; “3” = general need; “4” = high degree of need; and “5”= very needed.

### 2.2 Phase 2: Delphi expert consultation

Fifteen well‐known experts in DTC were invited and thirteen of them replied. Inclusion criteria of expert were as follows: (1) abundant professional knowledge in related fields; (2) Above 10 years working experience in thyroid surgery or nuclear medicine department; (3) intermediate certificate or above.

Consultation questionnaires were sent by email or face‐to‐face. Experts were asked to rate the importance of items on a numeric scale and give the modify advice for items. For each item, a five‐point Likert scale was devised to allow experts to evaluate the correlation between items and themes. Based on the expert opinions and group discussion results, items with low average score, poor relevance and ambiguous expression were deleted, and the similar items were merged. The consultation will be started again after 2 to 3 weeks. After the first round of Delphi, the research group revised items based on the expert opinions and then carried out the second round of Delphi.

In this study, two rounds of Delphi were conducted, the positive degree (positive coefficient >85%) and authority degree (authority coefficient 0.912) of 13 thyroid cancer experts were all good. Seven items were deleted, and nineteen items were modified after the first Delphi round. After the second Delphi round, three items were deleted, two items were modified and 1 item was added. At the same time, there is a greater consensus of expert opinions, so the consultation was terminated. A new consultation questionnaire with 43 items was formed after Delphi consultation.

### 2.3 Phase 3: Pilot testing

Feasibility and acceptability of the questionnaire were evaluated by pilot testing among 34 DTC patients treated with RAI therapy of the thyroid surgery and nuclear medicine department of one hospital. Respondents were welcome to put forward their views about this questionnaire. The questionnaire included a brief demographics questionnaire (e.g. age, sex, marital status, residence, educational level, and income level) and 43‐item pilot test version questionnaire. No respondents reported that items of the questionnaire were unclear or unintelligible, so the 43‐item pilot test version questionnaire was not modified following pilot testing.

### 2.4 Phase 4: Field testing

#### 2.4.1 Patients and procedures

The multi‐centre survey was administered in the thyroid surgery and nuclear medicine department of 3 hospital from August to December 2018. Inclusion criteria of patients: (1) above 18 years old; (2) were diagnosed with DTC (according to the pathology diagnosis result) (Haugen et al., 2016), had undergone thyroidectomy and were required to receive RAI therapy; (3) were fully conscious and able to read and communicate; (4) understood their conditions and were willing to participate; (5) did not have serious organic lesions. Exclusion criteria included any brain metastases of cancer, cognitive or intellectual impairment, history of psychosis and history of substance dependence.

The participants who agreed to participate in our study were asked to sign informed consents. Investigators would provide relevant information support to patients after they completed questionnaires. Initially, 240 patients met the criteria to serve as participants, but 10 refused to participate after being explained the purpose of this research to them. Therefore, 230 cases were collected, and the effective recovery of this study was 95.83%. Thirty respondents were reassessed two weeks later to test the questionnaire's retest reliability.

#### 2.4.2 Measures

Participants completed two consecutive questionnaires, a demographic and clinical characteristics questionnaire and the 43‐item pilot test version questionnaire. The demographic characteristics measure age, sex, residence, marital status, educational level and income level. Clinical variables explored the family and personal health histories of patients. Family history indicated thyroid disease. Personal history indicated types and times of operation, previous radiation exposure, complication and time of diagnosis.

### 2.5 Data collection

The investigators had been trained before the survey to ensure the quality of the investigation. Data were collected through face‐to‐face interviews within 30 min.

### 2.6 Data analysis

Data were entered into separate files by two independent researchers who had been trained and then verified and compared by EPI statistical software, version 3.1 (EPI‐3.1, The EpiData Association, Odense, Denmark). Afterwards, the data were conducted with Statistical Package for Social Sciences, version 21.0 (SPSS‐21.0, IBM). Descriptive statistics were used to describe participant characteristics. The exclusion criteria of item analysis were as follows: (1) the standard deviation <0.8; (2) critical ratio (*t)* <3.0; (3) the item correlation coefficient <0.40 or no statistical significance (Arraras et al., 2010). Construct validity was examined by exploratory factor analysis, which mainly includes principal component analysis (PCA) and varimax rotation. The Kaiser–Meyer–Olkin (KMO) measure and Bartlett's test of sphericity were used to ensure sampling adequacy for PCA. Kaiser's eigenvalues >1, scree plot and clinical interpretability were considered in extraction of factors (Shim et al., 2011). Reliability was examined by analysis of Cronbach's alpha, split‐half reliability and test‐retest reliability (Arraras et al., 2010).

## 3 RESULT

### 3.1 Patients characteristics

Table 1 presents socio‐demographic and clinical characteristics of participants (phase 3 and phase 4).

**TABLE 1:** Participant characteristics

	Phase 3 (*n*1 = 34)		Phase 4 (*n*2 = 230)	
	Number	%	Number	%
Age (year)				
18–39	17	50.00	81	35.22
40–64	16	47.10	141	61.30
≥65	1	2.90	8	3.48
Gender				
Male	12	35.30	58	25.22
Female	22	64.70	172	74.78
Marital status				
Single	1	2.90	17	7.39
Married	33	97.10	209	90.87
Widowed	0	0	4	1.74
Residence				
Rural	16	47.10	108	46.96
Urban	18	52.90	122	53.04
Education				
≤Middle school	16	47.10	107	46.52
High school	5	14.70	49	21.31
≥University/college	13	38.20	74	32.17
Income (RMB)				
<1,600	15	44.10	93	40.43
1600–3000	8	23.50	74	32.17
3000–5000	8	23.50	43	18.70
>5,000	3	8.80	20	8.70
Family history				
Yes	5	14.70	26	11.30
No	24	70.60	188	81.74
Dot know	5	14.70	16	6.96
Complication				
Yes	8	23.50	129	56.09
No	26	76.50	101	43.91
Times of surgery(time)				
1	32	94.10	208	90.43
≥2	2	5.90	22	9.57
RAI treatment				
Yes	1	2.90	61	26.52
No	33	97.10	169	73.48
Time of diagnosis(month)				
<6	33	97.10	165	71.74
≥6	1	2.90	65	28.26

Abbreviations: RAI, radioactive iodine; RMB, Renminbi.

In phase 4, mean age of participants was 44.53 ± 12.34 years, and the proportion of gender (men to women) nearly 1:3. The majority were married. Half of the sample reported that they lived in the countryside, with high school graduates and lower secondary education. Approximately 40% of the sample indicated that their income was lower than 1,600 RMB per month which merely achieves a minimal standard of living level in our city. Most of them did not have family history (thyroid disease) and previous radiation exposure, 56.1% had complication.

### 3.2 Validity and reliability analysis

#### 3.2.1 Item analysis

In this study, for each items, the standard deviation >1.0, critical ratio (*t)* >3.0 and item correlation coefficient >0.4, so following the items analysis, no item was deleted.

### 3.3 Validity analysis

#### 3.3.1 Content validity

Content Validity Index (CVI) is the most widely used index in quantitative evaluation (Polit & Beck, 2006). There are 2 kinds of CVI: Scale‐level CVI (S‐CVI) and item‐level CVI (I‐CVI). In this study, content validity was evaluated by 5 thyroid cancer specialists and 1 scaling expert, who were asked to rate the item correlation (from 1 to 4). The S‐CVI was 0.928, and I‐CVI was 0.929.

#### 3.3.2 Construct validity

Construct validity was examined by exploratory factor analysis. Sampling adequacy was confirmed by KMO measure (=0.97) and Bartlett's test of sphericity ($\chi^{2}$ = 6,104.395，*p <* . 001). Low factor loading Item 16 (≤0.4) was removed. Item 5, 14, 18, 27, 29, 39 and 40 had high factor loading (>0.4) on two common factors. The research group considered that item 27 and 29 are important components of the home‐based rehabilitation information theme, so these were temporarily retained. Item 5, 14, 16, 18, 39 and 40 were deleted after first EFA. Re‐analysis indicated KMO measure (=0.923) and Bartlett's test of sphericity ($\chi^{2}$ = 5,065.052，*p <* . 001). Double factor loading item 25, 26 and 28 were removed. The third time analysis generated a six‐factor structure, factor 6 only contain item 32 and 33, the number of item <3, so deleted. Using the Kaiser criterion, the fourth time analysis generated a five‐factor structure, which explained 61.9% of total variance and was a satisfactory solution from the Kaiser criterion, scree plot and clinical interpretability. The results are shown in Table 2. The five themes of the questionnaire were examination and operation information, radioactive iodine therapy information, psychosocial information, home‐based rehabilitation information and disease information. The correlation coefficient between each factor and the total score was 0.651 ~ 0.869, and the correlation coefficient among five factors was 0.384 ~ 0.677 (Table 3).

**TABLE 2:** Item factor categories and loading (*n* = 230)

Domains and items	Factor1	Factor2	Factor3	Factor4	Factor5
Examination and operation information					
Q11 Purpose of surgery	**0.788**	0.224	0.072	0.126	0.040
Q12 Surgical procedure	**0.748**	0.208	0.198	−0.081	0.123
Q6 Treatment options I can choice	**0.739**	0.066	0.144	0.184	0.239
Q8 The preparation before the examination	**0.718**	0.199	0.017	0.269	0.104
Q7 Purpose of examination	**0.672**	0.154	0.175	0.232	0.233
Q10The meaning of the examination results	**0.656**	0.213	0.149	0.110	0.179
Q13The effect of surgery	**0.645**	0.290	0.212	0.071	0.213
Q9 Adverse effects caused by examination	**0.638**	0.205	0.145	0.228	0.223
Q15The effect of surgery on the appearance of the operative area	**0.479**	0.091	0.206	0.335	0.090
Radioactive iodine therapy information					
Q20 Isolation environment for RAI therapy	0.228	**0.832**	0.037	0.194	0.056
Q24 How to reduce the adverse effect of radiation on myself and others after discharge	0.111	**0.760**	0.110	0.310	0.044
Q19 The procedure of RAI therapy	0.299	**0.754**	0.137	0.177	0.068
Q21 The preparation before RAI therapy (e.g. low iodine diet, drug withdrawal)	0.326	**0.606**	0.135	0.318	0.159
Q17 Purpose of RAI therapy	0.342	**0.591**	0.229	0.133	0.184
Q23 Adverse effects caused by RAI therapy	0.264	**0.575**	0.190	0.351	0.244
Psychosocial information					
Q36 How to remain optimistic and positive	0.128	0.134	**0.839**	0.264	0.116
Q37 How to communicate with my family better	0.193	0.210	**0.807**	0.170	0.126
Q35 How to deal with negative emotions (e.g. anxiety, depression, nervousness, fear, worry)	0.140	0.018	**0.783**	0.254	0.223
Q34 Effects of disease treatment on emotional and mental health	0.191	0.033	**0.714**	0.339	0.099
Q38 How to communicate with other patients	0.247	0.343	**0.680**	0.164	0.022
Home‐based rehabilitation information					
Q31 How to reexamine (time and frequency)	0.132	0.301	0.202	**0.646**	0.031
Q29 How to do rehabilitation exercises after discharge (neck and shoulders)	0.100	0.245	0.251	**0.640**	0.031
Q30 Long‐term adverse effects caused by treatment (sound impaired, hands and feet numbness, the discomfort of neck, shoulder and wound, scar)	0.361	0.261	0.219	**0.573**	0.173
Q27 What could I eat after discharge and what could not	0.068	0.283	0.081	**0.561**	0.172
Q42 The research progress of disease (e.g. new therapy, new rehabilitation techniques, new drugs)	0.154	0.108	0.286	**0.554**	0.023
Q41The impact of disease and treatment on work (or school)	0.255	0.080	0.331	**0.527**	0.089
Q22 How to deal with drug withdrawal reaction (fatigue, depression, weakness, swelling, loss of appetite and memory)	0.327	0.325	0.192	**0.400**	0.110
Disease information					
Q4 Whether the disease is hereditary	0.230	0.037	0.032	0.143	**0.765**
Q3 Morbidity, recurrence rate and mortality of disease	0.134	0.047	0.247	0.166	**0.759**
Q2 Possible causes of disease	0.267	0.143	0.108	0.179	**0.753**
Q1The meaning of terminology (e.g. thyroid cancer, thyroid cancer classification)	0.273	0.287	0.118	−0.198	**0.624**

Note: Bold indicates item own factor correlation higher than item correlation with the other factor of the questionnaire.

**TABLE 3:** The correlation coefficients between factors and questionnaire

	Disease information	Examination and operation information	Radioactive iodine therapy information	Home‐based rehabilitation information	Psychosocial information
Disease information	1	‐	‐	‐	‐
Examination and operation information	0.537**	1	‐	‐	‐
Radioactive iodine therapy information	0.419**	0.631**	1	‐	‐
Home‐based rehabilitation information	0.388**	0.597**	0.677**	1	‐
Psychosocial information	0.384**	0.497**	0.471**	0.646**	1
Total	0.651**	0.869**	0.821**	0.836**	0.742**

* Significance at *p* < .05.

** Significance at *p* < .01.

### 3.4 Reliability analysis

Internal consistency of the INQ‐DTC was optimal, with satisfactory Cronbach's alphas (0.945 for the total scale; range of for subscales=0.798 ~ 0.904). The total split‐half reliability was 0.822 and 0.749 ~ 0.873 for the domains, the test‐retest value was 0.984 for the overall score and 0.932 ~ 0.989 for the domains.

The final questionnaire items are presented in Box 1.

BOX 1 **The final 33 items questionnaire organized according to the theoretical framework**

**Table:** 

I need to know	Degree				
	Not at all	Low degree	General	High degree	Very
Survival information needs					
Disease information					
1. The meaning of terminology (e.g. thyroid cancer, thyroid cancer classification)					
2. Possible causes of disease					
3. Morbidity, recurrence rate and mortality of disease					
4. Whether the disease is hereditary					
Examination and operation information					
5. Treatment options I can choice					
6. Purpose of examination					
7. The preparation before the examination					
8. Adverse effects caused by examination					
9. The meaning of the examination results					
10. Purpose of surgery					
11. Surgical procedure					
12. The effect of surgery					
13. The effect of surgery on the appearance of the operative area					
Radioactive iodine therapy information					
14. Purpose of RAI therapy					
15. The procedure of RAI therapy					
16. Isolation environment for RAI therapy					
17. The preparation before RAI therapy (e.g. low iodine diet, drug withdrawal)					
18. Adverse effects caused by RAI therapy					
19. How to reduce the adverse effect of radiation on myself and others after discharge					
Home‐based rehabilitation information					
20. How to deal with drug withdrawal reaction (fatigue, depression, weakness, swelling, loss of appetite and memory)					
21. What could I eat after discharge and what could not					
22. How to do rehabilitation exercises after discharge (neck and shoulders)					
23. Long‐term adverse effects caused by treatment (sound impaired, hands and feet numbness, the discomfort of neck, shoulder and wound, scar)					
24. How to reexamine (time and frequency)					
25. The impact of disease and treatment on work (or school)					
26. The research progress of disease (e.g. new therapy, new rehabilitation techniques, new drugs)					
Interrelation and growth information needs					
Psychosocial information					
27. Effects of disease treatment on emotional and mental health					
28. How to deal with negative emotions (e.g. anxiety, depression, nervousness, fear, worry)					
29. How to remain optimistic and positive					
30. How to communicate with my family better					
31. How to communicate with other patients					
32. Overall satisfaction with the information you have received:	1	2	3	4	5
33. Do you wish to receive more information?	①Yes	②No			
If yes, please specify on which information?

## 4 DISCUSSION

The purpose of this study was to develop a questionnaire to assess the information needs of DTC patients with RAI therapy in mainland China. The development of INQ‐DTC was divided into four stage, this is, using literature review and semi‐structured interviews to generate the themes of the questionnaire and initial item bank, then through the Delphi method to determine the initial questionnaire, finally, the pilot testing and field testing were taken to assess the psychometric properties of this instrument, each of stage was aligned with best practice guidelines in questionnaire development^23^ and conducted rigorously to guarantee the scientificity of the questionnaire. Our findings indicated that INQ‐DTC is a reliable and effective tool for assessing the information needs of DTC patients in mainland China.

The INQ‐DTC focused on information needs of DTC patients in the specific treatment stage, which has been neglected by the majority of existing tools (Arraras et al., 2010; Dall'Armi et al., 2013; Huang et al., 2003). However, RAI therapy information is an important and specific component of information needs of DTC patients, which has been proved by our previous study (Zhang et al., 2019). Overall, the INQ‐DTC seems to help understand the needs of DTC patients and provide them with better clinical information support.

In terms of the content validity of the INQ‐DTC, which was evaluated by Delphi expert group. The S‐CVI value of the INQ‐DTC was 0.928, and 0.929 for the I‐CVI, which showed that all items of the INQ‐DTC have the ability to reflect the latent trait of DTC patients’ information needs appropriately and provided evidence of the good content validity.

In this study, construct validity of the INQ‐DTC was examined by exploratory factor analysis. As a result, the INQ‐DTC included information needs items in domains of disease information, examination and operation information, radioactive iodine therapy information, home‐based rehabilitation information and psychosocial information. Compared to the original version questionnaire, the final questionnaire reduced 19 items. After the EFA, items of surgical information from treatment information dimension and items from examination information dimensions were recombined into the examination and operation information dimension, items of RAI therapy information from treatment information dimension reconstituted into a new dimension named radioactive iodine therapy information dimension. The reason might be the RAI treatment usually performed at least one month later after the surgery, and patients are required to stay in an isolation ward which is completely different from the ordinary ward (Haugen et al., 2016). Therefore, RAI therapy information had become a specific and independent dimension in the information needs of DTC patients with RAI therapy. We found that the social support information dimension and the self‐growth information dimension were combined into the psychosocial information dimension. Five factors of the questionnaire explained 61.9% of total variance and all indicators were satisfactory, which indicated that the INQ‐DTC has a reasonable structure and a satisfactory construct validity.

The internal consistency of INQ‐DTC was examined by Cronbach's alphas coefficient, split‐half reliability and test‐retest reliability in this study. The total Cronbach's alpha coefficient was 0.945 and 0.798 ~ 0.904 for the domains, the total split‐half reliability was 0.822 and 0.749 ~ 0.873 for the domains. These findings indicated that the INQ‐DTC has satisfactory internal consistency reliability (Arraras et al., 2010), could reflect the information needs of DTC patients with RAI therapy sufficiently. The test‐retest value was 0.984 for the overall score and 0.932 ~ 0.989 for the domains, denoting high stability of the INQ‐DTC over time. The results above indicated that the INQ‐DTC was a reliable and stable tool for evaluating the information needs of DTC patients with RAI therapy and could be promoted.

INQ‐DTC is of guiding significance in nursing of DTC patients. Understanding the information needs of patients is the basis of effective nurse‐patient communication. For nurses, INQ‐DTC could provide a scientific and reliable specific assessment tool for them. It could help nurses understand and evaluate patients’ information needs comprehensively and identify which is the first information needs of patients in a quick way. This tool could also be used to evaluate the effectiveness of health education provided by nurses. Based on the above, INQ‐DTC could provide reference for precise health education and improve the work efficiency and care quality of clinical nurses. This questionnaire could also be used in further multi‐centre investigation program. For patients, INQ‐DTC could guide them to think their information needs more deeply, stimulate their unrealized implicit information need and help them express their explicit information needs better, so as to promote the effectiveness of communication between patients and nurses.

There are several limitations in this study should be noted. First, we have insufficient sample size in this study, which might diminish the veracity of factor analysis results. In future studies, larger samples are needed to validate the INQ‐DTC. Second, because of the lack of information needs assessment tools for DTC patients, we could not find an appropriate “gold standard” scale for criterion validity test in this study, and further studies on the criterion validity of the scale are needed.

## 5 CONCLUSION

As far as we know, INQ‐DTC is the first specificity instrument for measuring information needs of DTC patients with RAI therapy in mainland China. The results show that all indicators of the INQ‐DTC met the measurement standards, which is a validity and stability tool for assessing information needs. Besides, the INQ‐DTC may help to identify areas for information support development which could ultimately improve the individual care and information support received by DTC patients with RAI therapy.

## 6 RELEVANCE TO CLINICAL PRACTICE

The development of the INQ‐DTC holds promise of identifying information needs of DTC patients with RAI therapy accurately, which could guide clinical information support and ultimately improve the individual care received by patients.

## 7 INFORMED CONSENT

All participants signed informed consent form at study onset, according to the recommendations of the Research Ethics Committee at Zhengzhou University.

## CONFLICT OF INTEREST

The authors declare that they have no conflicts of interest.

## AUTHOR CONTRIBUTION

Limei Li, Tingting Zhu, Qiaoqiao Gao, Hui Chen, Ling Ma, Jiayin Li and Zichen Wang: Performance of material preparation, data collection, and analysis. Dongling Liu: Mainly in the charge of funding acquisition and article review. Jing Zhang: First draft of the manuscript. All authors contributed to the study conception and design, commented on previous versions of the manuscript, read and approved the final manuscript.

## ETHICAL APPROVAL

All procedures performed in studies involving human participants were in accordance with the ethical standards of the institutional and/or national research committee and with the 1964 Helsinki declaration and its later amendments or comparable ethical standards.

## Data Availability

Some or all data, models, or code generated or used during the study are available from the corresponding author by request.

## References

[nop2925-bib-0001] Alderfer, C. P. (1969). An empirical test of a new theory of human needs. Organizational Behaviour and Human Performance, 2(4), 142–175. 10.1016/0030-5073(69)90004-X

[nop2925-bib-0002] Arraras, J. I. , Greimel, E. , Sezer, O. , Chie, W. , Bergenmar, M. , Costantini, A. , Young, T. , Vlasic, K. K. , & Velikova, G. (2010). An international validation study of the EORTC QLQ‐INFO25 questionnaire: An instrument to assess the information given to cancer patients. European Journal of Cancer, 46(15), 2726–2738. 10.1016/j.ejca.2010.06.118 20674333

[nop2925-bib-0003] Au, A. , Lam, W. W. , Kwong, A. , Suen, D. , Tsang, J. , Yeo, W. , Suen, J. , Ho, W. M. , Yau, T. K. , Soong, I. , Wong, K. Y. , Sze, W. K. , Ng, A. , Girgis, A. , & Fielding, R. (2011). Validation of the Chinese version of the short‐form supportive care needs survey questionnaire (SCNS‐SF34‐C). Psycho‐Oncology, 20(12), 1292–1300. 10.1002/pon.1851 22114044

[nop2925-bib-0004] Boyes, A. , Girgis, A. , & Lecathelinais, C. (2009). Brief assessment of adult cancer patients' perceived needs: Development and validation of the 34‐item Supportive Care Needs Survey (SCNS‐SF34). Journal of Evaluation in Clinical Practice, 15(4), 602–606. 10.1111/j.1365-2753.2008.01057.x 19522727

[nop2925-bib-0005] Bray, F. , Ferlay, J. , Soerjomataram, I. , Siegel, R. L. , Torre, L. A. , & Jemal, A. (2018). Global cancer statistics 2018: GLOBOCAN estimates of incidence and mortality worldwide for 36 cancers in 185 countries. CA: A Cancer Journal for Clinicians, 68(6), 394–424. 10.3322/caac.21492 30207593

[nop2925-bib-0006] Cabanillas, M. E. , McFadden, D. G. , & Durante, C. (2016). Thyroid cancer. Lancet, 388(10061), 2783–2795. 10.1016/S0140-6736(16)30172-6 27240885

[nop2925-bib-0007] Chen, W. , Zheng, R. , Baade, P. D. , Zhang, S. , Zeng, H. , Bray, F. , Jemal, A. , Yu, X. Q. , & He, J. (2016). Cancer statistics in China, 2015. CA: A Cancer Journal for Clinicians, 66(2), 115–132. 10.3322/caac.21338 26808342

[nop2925-bib-0008] Cossich, T. , Schofield, P. , & McLachlan, S. A. (2004). Validation of the cancer needs questionnaire (CNQ) short‐form version in an ambulatory cancer setting. Quality of Life Research, 13(7), 1225–1233. 10.1023/B:QURE.0000037496.94640.d9 15473501

[nop2925-bib-0009] Dall'Armi, L. , Simpson, G. K. , Forstner, D. , Simpson, T. , Roydhouse, J. K. , & White, K. J. (2013). The information needs of patients with head and neck cancer and their caregivers: A short report of instrument development and testing. Applied Nursing Research, 26(1), 40–44. 10.1016/j.apnr.2012.08.001 23218958

[nop2925-bib-0010] Fang, S. , Cheng, H. , & Lin, C. (2018). Validation of the modified Chinese Cancer Survivor's Unmet Needs (CaSUN‐C) for women with breast cancer. Psychooncology, 27(1), 236–242. 10.1002/pon.4499 28699657

[nop2925-bib-0011] Goldfarb, M. , & Casillas, J. (2014). Unmet information and support needs in newly diagnosed thyroid cancer: Comparison of adolescents/young adults (AYA) and older patients. Journal of Cancer Survivorship, 8(3), 394–401. 10.1007/s11764-014-0345-7 24570216

[nop2925-bib-0012] Haugen, B. R. , Alexander, E. K. , Bible, K. C. , Doherty, G. M. , Mandel, S. J. , Nikiforov, Y. E. , Pacini, F. , Randolph, G. W. , Sawka, A. M. , Schlumberger, M. , Schuff, K. G. , Sherman, S. I. , Sosa, J. A. , Steward, D. L. , Tuttle, R. M. , & Wartofsky, L. (2016). 2015 American thyroid association management guidelines for adult patients with thyroid nodules and differentiated thyroid cancer: The American Thyroid association guidelines task force on thyroid nodules and differentiated thyroid cancer. Thyroid, 26(1), 1–133. 10.1089/thy.2015.0020 26462967 PMC4739132

[nop2925-bib-0013] Heckel, L. , Fennell, K. M. , Reynolds, J. , Osborne, R. H. , Chirgwin, J. , Botti, M. , Ashley, D. M. , & Livingston, P. M. (2015). Unmet needs and depression among carers of people newly diagnosed with cancer. European Journal of Cancer, 51(14), 2049–2057. 10.1016/j.ejca.2015.06.129 26208461

[nop2925-bib-0014] Heynsbergh, N. , Botti, M. , Heckel, L. , & Livingston, P. M. (2018). Caring for the person with cancer: Information and support needs and the role of technology. Psycho‐Oncology, 27(6), 1650–1655. 10.1002/pon.4722 29624783 PMC6001456

[nop2925-bib-0015] Hodgkinson, K. , Butow, P. , Hunt, G. E. , Pendlebury, S. , Hobbs, K. M. , Lo, S. K. , & Wain, G. (2007). The development and evaluation of a measure to assess cancer survivors' unmet supportive care needs: The CaSUN (Cancer Survivors' Unmet Needs measure). Psycho‐Oncology, 16(9), 796–804. 10.1002/pon.1137 17177268

[nop2925-bib-0016] Huang, X. W. , Zhang, Y. , Wang, X. L. , Lv, B. X. , & Li, Y. J. (2003). Information needs of cancer patients‐Development and evaluation of Information Preference Questionnaire for Cancer Patients. Chinese Mental Health Journal, 11, 750–753.

[nop2925-bib-0017] Husson, O. , Thong, M. S. , Mols, F. , Oerlemans, S. , Kaptein, A. A. , & van de Poll‐Franse, L. V. (2013). Illness perceptions in cancer survivors: What is the role of information provision? Psycho‐Oncology, 22(3), 490–498. 10.1002/pon.3042 22307579

[nop2925-bib-0018] Johnson, C. , Aaronson, A. , & Blazeby, J. M. (2011). Guidelines for developing questionnaire modules (4th edn Vol. 7): EORTC Quality of Life Group.

[nop2925-bib-0019] Kim, S. G. , Paeng, J. C. , Eo, J. S. , Shim, H. K. , Kang, K. W. , Chung, J. K. , Lee, M. C. , & Lee, D. S. (2010). Behavior and awareness of thyroid cancer patients in Korea having non‐hospitalized low‐dose radioiodine treatment with regard to radiation safety. Nuclear Medicine and Molecular Imaging, 44(4), 267–272. 10.1007/s13139-010-0057-5 24899963 PMC4042913

[nop2925-bib-0020] Kuenzel, U. , Monga, S. T. , Schroth, S. , Huebner, J. , & Herth, N. (2018). Evaluation of the quality of online information for patients with rare cancers: Thyroid cancer. Journal of Cancer Education, 33(5), 960–966. 10.1007/s13187-017-1173-z 28120139

[nop2925-bib-0021] Lamers, R. E. , Cuypers, M. , Husson, O. , de Vries, M. , Kil, P. J. , Ruud, B. J. , & van de Poll‐Franse, L. V. (2016). Patients are dissatisfied with information provision: Perceived information provision and quality of life in prostate cancer patients. Psycho‐Oncology, 25(6), 633–640. 10.1002/pon.3981 26403417

[nop2925-bib-0022] Morley, S. , & Goldfarb, M. (2015). Support needs and survivorship concerns of thyroid cancer patients. Thyroid, 25(6), 649–656. 10.1089/thy.2015.0032 25809716

[nop2925-bib-0023] Nickel, B. , Howard, K. , Brito, J. P. , Barratt, A. , Moynihan, R. , & McCaffery, K. (2018). Association of preferences for papillary thyroid cancer treatment with disease terminology: A discrete choice experiment. JAMA Otolaryngol Head Neck Surg, 144(10), 887–896. 10.1001/jamaoto.2018.1694 30140909 PMC6233835

[nop2925-bib-0024] Owen‐Smith, A. , Coast, J. , & Donovan, J. (2010). Are patients receiving enough information about healthcare rationing? A qualitative study. Journal of Medical Ethics, 36(2), 88–92. 10.1136/jme.2009.033241 20133402

[nop2925-bib-0025] Polit, D. F. , & Beck, C. T. (2006). The content validity index: Are you sure you know what's being reported? critique and recommendations. Research in Nursing & Health, 29(5), 489–497. 10.1002/nur.20147 16977646

[nop2925-bib-0026] Sawka, A. M. , Brierley, J. D. , Tsang, R. W. , Rotstein, L. , Ezzat, S. , & Goldstein, D. P. (2016). Unmet information needs of low‐risk thyroid cancer survivors. Thyroid, 26(3), 474–475. 10.1089/thy.2015.0569 26671279

[nop2925-bib-0027] Sawka, A. M. , Carty, S. E. , Haugen, B. R. , Hennessey, J. V. , Kopp, P. A. , Pearce, E. N. , Sosa, J. A. , Tufano, R. P. , & Jonklaas, J. (2018). American thyroid association guidelines and statements: Past, present, and future. Thyroid, 28(6), 692–706. 10.1089/thy.2018.0070 29698130

[nop2925-bib-0028] Shim, E. J. , Lee, K. S. , Park, J. H. , & Park, J. H. (2011). Comprehensive needs assessment tool in cancer (CNAT): The development and validation. Supportive Care in Cancer, 19(12), 1957–1968. 10.1007/s00520-010-1037-0 21076926

[nop2925-bib-0029] Siegel, R. L. , Miller, K. D. , & Jemal, A. (2019). Cancer statistics, 2019. CA: A Cancer Journal for Clinicians, 69(1), 7–34. 10.3322/caac.21551 30620402

[nop2925-bib-0030] Ulloa, J. G. , Hemmelgarn, M. , Viveros, L. , Odele, P. , Feldman, N. R. , Ganz, P. A. , & Maggard‐Gibbons, M. (2015). Improving breast cancer survivors' knowledge using a patient‐centered intervention. Surgery, 158(3), 669–675. 10.1016/j.surg.2015.03.056 26032819 PMC4820246

[nop2925-bib-0031] Zhang, J. , Li, L. M. , Li, J. Y. , Wang, Z. C. , & Liu, D. L. (2019). Investigation on health information acquisition and demand status of patients with thyroid cancer. Chinese Nursing Research, 33, 3872–3878. 10.12102/j.issn.1009-6493.2019.22.010

